# 

**Published:** 1916-11-18

**Authors:** 


### CONTENTS.

PAGE

**Medical Degrees and Diplomas** ... ... ... 129

**Hospital Managers in Municipal Office** ... 130

**Hospital and Institutional News** ... ... ... 131

A British Red Cross Unit for Rumania—More Women Medical Officers for the Army—Sir Sam Hughes's Allegations About the Treatment of Wounded Canadians—Two Vacant Hospital Secretaryships—The Diphtheria Scare Again—Welsh National Memorial Replies to Criticism—The Redditch Smallwood Hospital—Infant Consultations at Queen Charlotte's Lying-in Hospital—A Question of Principle at Dunfermline—The Dundee Asylum Impasse—Sanatorium Buildings at £12 per Bed—Miss Beckett Denison's Legacy to the Doncaster Royal Infirmary—Gifts in Kind to Hospitals—Short Shrift for Pro-Germans—An Official National Insurance Handbook—A Godalming Doctor Who Attended Wellington—The Cocaine Committee—" Inasmuch "—Raiding the Poultry Farmer—The Fallacy of the Old Debt—War Charities—The Length of Appeals—The Evolution of the Bath-Chair—Silhouettes in Cross-Stitch—This Week's Drug Market—To Our Readers.

**Moral** ... ... ... ... ... ... ... ... 137

Its First Reinforcement.

**The Local Incidence of Infectious Diseases** ... 138

**The Hospitals of the United States** ... ... 139

The Future is Youth's Freehold.

**Gifts in Kind** ... .. ... ... ... ... 140

Their Valuation in Hospital Accounts.

PAGE

**National Insurance Act Developments...** ... 141

The Need for Businesslike Methods.

**The Matrons' and Sisters' Department** ... 143

State Registration: The Central Committee and the College of Nursing.

Text of the Correspondence.

Round the Hospitals.

Miss Rundle's Assistant—Applications to Register—Hastening the Decision—Prospective College Meetings—" Curbing " Florence Nightingale—Miss Haughton's Condition.

**Patriotism and the Nurse-Training Schools** ... 148

X.—Leicester, Sheffield, Salisbury.

**The Modified Pay System** ... ... ... ... 149

Patients' Payments and Contributions.

**Legal Notes for Institutional Workers** ... 151

**Passing Events and Topics** ... ... ... ... 151

**Gifts to Hospitals** ... ... ... .. ... 152

**Matrons' and Sisters' Appointments** ... ... 152

**Parliament and the Profession** ... ... ... 152

**The Book Market** ... ... ... ... ... 152

**The Institutional Worker** ... .. (Suppl.) 1

The Supply of Comforts for Wounded and Warriors.

Question for November.

Enquiries and Answers.

The Sick and in Need.

Miscellaneous.

